# Effects of exercise in a fed or fasted state on mitochondrial dynamics regulators in the skeletal muscle of recreationally active males

**DOI:** 10.1113/EP093336

**Published:** 2026-08-06

**Authors:** Elya J. Ritenis, Christos G. Stathis, Matthew B. Cooke, Stephen Keenan, Andrew Philp, Donny M. Camera

**Affiliations:** ^1^ Department of Health Sciences and Biostatistics Swinburne University of Technology Melbourne Victoria Australia; ^2^ Centre for Healthy Ageing Centenary Institute Sydney New South Wales Australia; ^3^ Faculty of Medicine and Health, Charles Perkins Centre University of Sydney Sydney New South Wales Australia; ^4^ College of Sport, Health and Engineering Victoria University Melbourne Victoria Australia; ^5^ Institute for Health and Sport Victoria University Melbourne Victoria Australia; ^6^ Sport, Performance, and Nutrition Research Group, School of Allied Health, Human Services and Sport La Trobe University Melbourne Victoria Australia; ^7^ School of Sport, Exercise and Rehabilitation Sciences University of Technology Sydney Sydney New South Wales Australia

**Keywords:** exercise, mitochondrial dynamics, skeletal muscle

## Abstract

Exercise and nutritional modulation favourably alter mitochondrial quantity and quality in skeletal muscle. Mitochondrial dynamics, the coordination of fission and fusion events, are poised to mediate key aspects of organelle adaptation that arise from exercise. However, the molecular basis by which exercise affects mitochondrial dynamics remains poorly understood. The objective of this work was to further elucidate the signalling response of mitochondrial dynamics regulators to exercise and explore the synergistic potential to combine exercise and nutritional modulation. In a randomized crossover design, eight healthy, recreationally active men (age 25.8 ± 5.3 years, BMI 24.4 ± 1.2 kg/m^2^, ${\dot{V}}_{O_{2}\mathrm{peak}}$ 39.2 ± 5.7 ml/kg/min) performed a 1‐h bout of workload‐matched aerobic exercise on a cycle ergometer at 50–70% of *W*_max_, either in a fasted or a fed (i.e., following a carbohydrate rich breakfast) state. Gas exchange was measured throughout, and blood samples were collected intermittently. Vastus lateralis muscle biopsies were collected pre‐, post‐ and 3 h post‐exercise. Western blotting was performed on cytosolic and mitochondrial fractions. Fasted exercise was accompanied by increased cytosolic acetyl‐CoA carboxylase Ser79 phosphorylation (*P* ≤ 0.001), an effect not observed in the fed group (*P* > 0.05). The subcellular location of mitochondrial fission effector dynamin‐related protein 1 (DRP1) was unchanged (*P* > 0.05) following exercise. However, group differences (i.e., Fed vs. Fasted) in DRP1 subcellular localization and phosphorylation at residues Ser616 and Ser637 were observed post‐exercise. Substrate availability may potentially influence the mitochondrial dynamics signalling response in skeletal muscle to acute aerobic exercise.

## Abstract (highlights)

- **What is the central question of this study?**
  This study investigates the mitochondrial dynamics signalling response to acute aerobic exercise in recreationally active males and examines the potential for fasting to enhance this signalling response when synergistically combined with exercise.
- **What is the main finding and its importance?**
  Increased cytosolic ACC phosphorylation was observed following fasted exercise, suggesting a distinct subcellular signalling response characterised by increased stress signalling. This was accompanied by post‐exercise group differences in the phosphorylation and subcellular localization of DRP1, indicating that substrate availability (i.e., fasting) may influence the mitochondrial dynamics signalling response of skeletal muscle to acute aerobic exercise.

## 1 INTRODUCTION

The mitochondrial reticulum, a distinct three‐dimensional tubular network formed by the organelle in skeletal muscle, varies between different mitochondrial subpopulations and muscle fibre types (Bleck et al., 2018; Glancy et al., 2015). Mitochondrial morphology is shaped by a dynamic flux of fusion and fission events, culminating in ongoing architectural remodelling of the mitochondrial reticulum (Sabouny & Shutt, 2020; Tilokani et al., 2018). Mitochondrial fusion enhances reticulum branching and connectivity by incorporating separate and distinct mitochondria into a single structure (Sabouny & Shutt, 2020; Tilokani et al., 2018).

Conversely, mitochondrial fission promotes fragmentation of the mitochondrial reticulum by dividing mitochondria into separate, distinct structures (Sabouny & Shutt, 2020; Tilokani et al., 2018). Given the close association between mitochondrial form and function, acute alterations in reticulum morphology enable mitochondria to optimize organelle bioenergetics (Tilokani et al., 2018). This is of relevance under conditions of metabolic stress, such as exercise, where energy demand can increase 100‐fold (Hargreaves & Spriet, 2020). Previous studies in humans and murine models have highlighted that acute bouts of aerobic or resistance exercise upregulate protein and gene expression of targets related to fission signalling (Coronado et al., 2018; Ding et al., 2010; Kitaoka et al., 2015; Kruse et al., 2016; Moore et al., 2019; Schwalm et al., 2017). Indeed, previous murine research using a novel peptide inhibitor to prevent pro‐fission protein interactions has highlighted that the acute mitochondrial fission response can impact exercise capacity (Coronado et al., 2018). Additionally, fission has been implicated in sequestering the mitochondria targeted for exercise‐induced mitophagy (Laker et al., 2017).

The mechanistic basis for a bout of exercise to elicit mitochondrial fission remains poorly understood. Previous in vitro work has highlighted the potential for mitochondrial fission to occur in an AMP‐activated protein kinase (AMPK)‐dependent fashion (Seabright et al., 2020; Toyama et al., 2016) through direct phosphorylation of mitochondrial fission factor (MFF) at Ser146 (Seabright et al., 2020). Once recruited by adaptors such as MFF, dynamin‐related protein 1 (DRP1) forms a complex around the targeted mitochondria, constricting it in a GTP‐dependent manner until severance (Kalia et al., 2018; Kamerkar et al., 2018). Nevertheless, the in vivo relevance of AMPK‐mediated mitochondrial fission in the context of exercise is yet to be established. Further, it is unknown how varying degrees of cellular stress may alter the mitochondrial dynamics signalling response with exercise. Stocks and colleagues have previously demonstrated that a given bout of aerobic exercise in the fasted state (i.e. following an overnight fast) enhances AMPK^Thr172^ phosphorylation (indicative of its activation), when compared to performing the same exercise bout in the unfasted (‘fed’) state (Stocks et al., 2019). The metabolic perturbations imposed by exercise and fasting have both independently been shown to increase AMPK activation (Cantó et al., 2010; Chen et al., 2003). In this regard, elevated AMPK activation suggests that there is a synergistic effect of fasting and exercise that, when superimposed, can acutely enhance metabolic stress.

While it is presumptuous to assume that upregulation of AMPK signalling translates to a greater induction of more mitochondrial processes (such as dynamics), previous studies have demonstrated how facets of other mitochondrial processes, such as biogenesis, are malleable to manipulation of exercise intensity or volume to augment the exercise response (Egan et al., 2010; Fiorenza et al., 2018; Gibala et al., 2006; Serpiello et al., 2012; Skovgaard et al., 2016). Importantly, factors relating to nutrition or stress associated with nutritional deprivation (i.e., fasting) have also been shown to manipulate the acute and chronic effects of exercise on the other mitochondrial quality control processes (Bartlett et al., 2013; Camera et al., 2015; Kitaoka et al., 2016; Psilander et al., 2013; Schwalm et al., 2017). Elevated AMPK signalling during fasted exercise could potentially enhance phosphorylation of downstream substrates such as Parkin^Ser108^ (Hung et al., 2021) and MFF^Ser146^ (Seabright et al., 2020), providing a molecular basis for induction of mitochondrial dynamics and mitophagy processes.

The overall objective of this study was to investigate exercise‐induced mitochondrial dynamic signalling response following a single bout of aerobic exercise commenced in either a fed or a fasted state. It was hypothesised that the enhanced cellular stress during fasted exercise would underpin augmented mitochondrial fission signalling.

## 2 METHODS

### 2.1 Ethical approval

This research study and all experimental procedures were approved by the Swinburne University of Technology Human Research Ethics Committee (Ref: 2022635710988). The study conformed to the policy statement regarding the use of human participants in the latest revision of the *Declaration of Helsinki*, except for registration in a database. Prior to any experimentation informed consent was obtained from participants.

### 2.2 Participants

Eight recreationally active, healthy men aged 20–36 years and with a BMI between 18.5 and 24.9 kg/m^2^ were recruited for this study (Table 1). Participants were briefed on all procedures involved in the study and any potential risks and informed of their right to withdraw before signing a consent form. The following inclusion criteria were applied: (1) healthy male aged 20–36 years, (2) BMI 18.5–24.9 kg/m^2^, (3) recreationally active, (4) non‐smoker, and (5) omnivore.

**TABLE 1:** Baseline characteristics of study participants.

Characteristic	Value (*n* = 8)
Age (years)	25.8 ± 5.3
Height (cm)	178.7 ± 11.1
Weight (kg)	78.1 ± 10.1
BMI (kg/m^2^)	24.4 ± 1.2
${\dot{V}}_{O_{2}\mathrm{peak}}$ (ml/kg/min)	39.2 ± 5.7

*Note*: Data are presented as means ± SD (*n* = 8).

### 2.3 Preliminary testing

Following the recording of height and weight, participants performed an aerobic fitness test (${\dot{V}}_{O_{2}\mathrm{peak}}$) on a cycle ergometer until exhaustion (Excalibur; Lode, Groningen, The Netherlands). The test began with an initial warmup of 100 W for 3 min after which the workload increased in 35 W increments every 3 min. Volume of oxygen (${\dot{V}}_{O_{2}}$) consumption and volume of carbon dioxide (${\dot{V}}_{CO_{2}}$) production were then recorded using open‐circuit spirometry (TrueOne 2400; Parvo Medics, Salt Lake City, UT, USA). Heart rate (HR) was continually monitored for the duration of the test (H10; Polar, Kempele, Finland). Ratings of perceived exertion (RPE) were determined using the 6–20 Borg scale during the final 15 s of each 3 min stage. The following criteria were used to determine if a participant achieved their ${\dot{V}}_{O_{2}\mathrm{peak}}$: (1) respiratory exchange ratio (${\dot{V}}_{CO_{2}}$/${\dot{V}}_{O_{2}}$) > 1.1; (2) HR within 10 beats of the age‐predicted (220 − age) max; or (3) plateau in oxygen consumption with increasing workload. Participant workload max (*W*_max_) was determined as the work rate of the last completed stage, plus the fraction of time spent in the final stage multiplied by the stage increment (35 W).

### 2.4 Experimental trials

Participants performed two experimental trials in a randomized, counterbalanced, crossover design. Given that the study design necessitated that participants consume food, it was not possible to blind participants or researchers. Participants were instructed to refrain from alcohol for 72 h, exercise for 48 h and caffeine for 24 h. Participants were provided with a standardized diet for a 24 h period preceding both trials (energy contribution: carbohydrates 61%, fat 18%, and protein 21%). Participants arrived in the exercise laboratory at 07.00 h after an overnight fast (∼12 h), with participants permitted to consume water ad libitum during the fasting period. Immediately after arriving at the lab, baseline blood samples were obtained, and participants were randomly allocated to one of two dietary treatments in a balanced, cross‐over design:

1. Fed: participants received a standardized carbohydrate rich breakfast to consume within 15 min. The breakfast meal comprised cornflakes (0.9 g/kg), semi‐skimmed milk (3.9 mL/kg), toasted wholemeal bread (1.1 g/kg), strawberry jam (0.3 g/kg) and orange juice (3.2 mL/kg), and had the following macronutrient composition: 75% carbohydrates, 10% fat and 15% protein.
2. Fasted: participants remained in the fasted state.

Following this, participants rested for a further 2 h, before a muscle biopsy from the vastus lateralis was taken. Participants then performed a 1 h bout of exercise on the cycle ergometer (Lode Excalibur) at an intensity of 50%–70% of their *W*_max_.

Participants began at a workload of 70% *W*_max_, which was adjusted as required and counterbalanced to the subsequent trial to ensure participants could complete the hour‐long exercise bouts. Spirometry was used to measure respiratory variables (MOXUS Metabolic Cart; AEI Technologies, Bastrop, TX, USA) throughout the exercise bout. RPE (6–20 Borg scale) and HR (Polar H10) were also monitored at 15 min intervals throughout exercise. Immediately after finishing exercise, another muscle biopsy was taken. Participants then rested for 3 h, before the final muscle biopsy was taken. Venous blood sampling occurred at 20, 40, 60, 120, 135, 150, 165, 180, 200, 220, 240, 300 and 360 min following the baseline blood sample. Total water consumption across the trial was recorded to account for any possible discrepancies. Participants returned to the laboratory approximately 9–14 days later to complete the second trial. All biopsies were taken from the opposite leg during the second trial.

### 2.5 Muscle biopsies

Muscle biopsies for each trial were obtained from three separate incision sites on the vastus lateralis using a Bergström needle that had been modified to apply suction with a syringe as previously described (Camera et al., 2012). Local anaesthetic (1% lidocaine) was injected into the biopsy site. Muscle samples were blotted with filter paper to remove excess blood and connective tissue, then flash‐frozen in liquid nitrogen, before storage at −80°C until further analysis.

### 2.6 Blood sampling

Blood samples were collected into EDTA treated vacutainers (BD, Wokingham, UK) and then centrifuged at 1250*g* for 10 min at 4°C. The separated plasma was then aliquoted into Eppendorf tubes (Eppendorf, Hamburg, Germany) before undergoing analysis for glucose and lactate levels (2950D Biochemistry Analyzer; YSI, Yellow Springs, OH, USA). Plasma samples were stored at −80°C until further analysis.

### 2.7 Mitochondrial and cytosolic fractionation

Using modified previously published methods (Peters et al., 1998), muscle biopsy samples were homogenized and the enriched mitochondrial and cytosolic fractions isolated. Muscle biopsies were first processed on dry ice using The Cellcrusher (Cellcrusher, Schull, Ireland) to pulverize the samples into a fine powder. To ensure consistency in the volume of buffer used per sample, 50 mg aliquots of powdered tissue were first weighed out over dry ice and recorded. The powdered muscle biopsy sample was first homogenized by hand in a Dounce homogenizer with 20 times volume of an ice‐cold buffer (pH 7.5) composed of: 100 mM KCl, 40 mM Tris–HCl pH 6.8, 10 mM Tris–HCl pH 8.8, 5 mM MgSO_4_, 1 mM EDTA, 1 mM ATP. The homogenate was then transferred to an ice‐cold 15 mL conical centrifuge tube and centrifuged at 800 *g* for 10 min at 4°C. The resulting supernatant was transferred to an ice‐cold 2 mL Eppendorf tube and centrifuged (Fresco 21, Thermo Fisher Scientific, Waltham, MA, USA) at 14,000 *g* for 10 min at 4°C. Following this, the supernatant (crude cytosolic fraction) was centrifuged again at 14,000 *g* for 10 min at 4°C, and the resulting supernatant (cytosolic fraction) was then transferred to an ice‐cold 2 mL Eppendorf tube. The pellet (crude mitochondrial fraction) was resuspended twice in buffers (pH 7.5) composed of 100 mM KCl, 40 mM Tris–HCl pH 6.8, 10 mM Tris–HCl pH 8.8, 1 mM MgSO_4_, 0.1 mM EDTA, 0.25 mM ATP and centrifuged at 7000 *g* for 10 min at 4°C. The first of these buffers was prepared in 1% bovine serum albumin (BSA) and the second was prepared without. Lastly, the resulting pellet (mitochondrial fraction) was resuspended in an ice‐cold buffer (pH7.4) composed of: 220 mM sucrose, 70 mM mannitol, 10 mM Tris–HCl pH 8.8, 1 mM EDTA. All buffers were supplemented with the cOmplete protease (Roche, Basel, Switzerland) and PhosSTOP phosphatase (Roche) inhibitor cocktails. Enrichment of mitochondrial and cytosolic fractions was confirmed by western blotting for the content of the mitochondrially ubiquitous voltage‐dependent anion channel (VDAC) and cytosolic glyceraldehyde 3‐phosphate dehydrogenase (GAPDH) proteins, respectively (described below).

### 2.8 Western blotting

The protein concentration of mitochondrial and cytosolic enriched fractions was determined using a DC protein assay (Bio‐Rad Laboratories, Hercules, CA, USA). Samples were then boiled for 5 min at 95°C in 4× Laemmli sample buffer (Bio‐Rad) and 2‐

Mercaptoethanol (Sigma‐Aldrich, St Louis, MO, USA). Ten micrograms of protein was loaded for mitochondrial and cytosolic enriched fractions and separated by SDS‐PAGE using 26 lane 4–15% Criterion TGX Precast Midi Protein Gels (Bio‐Rad) at a constant of 110 V for approximately 1 h. Proteins were then transferred onto 0.45 µm nitrocellulose membranes (Bio‐Rad) via a wet transfer performed at a constant of 100 V for 1 h. Following this, membranes were stained with Ponceau S (Sigma‐Aldrich) to assess for consistent protein loading and transfer. Membranes probing for total and phosphorylated proteins were blocked in 3% dry‐milk or 3% BSA with Tris‐buffered saline and tween (TBST) for 1 h at room temperature, respectively. After blocking, membranes were rinsed 3 times in TBST for 5 min and then incubated overnight in primary antibody, at 4°C. After incubation in primary antibody, membranes were rinsed 3 times in TBST for 5 min and then incubated in secondary antibody for 1 h, at room temperature. Lastly, membranes were rinsed 3 times in TBST for 5 min and then antibody detection was conducted using an enhanced chemiluminescence horseradish peroxidase substrate detection kit (Millipore, Watford, UK). Imaging and band quantification were performed using the ChemiDoc Touch (Bio‐Rad) and ImageLab Software (Bio‐Rad), respectively. All western blot data were normalized to the total protein loaded into each lane, as determined by the Ponceau S (Sigma‐Aldrich) stain. All primary antibodies were used at a concentration of 1:1000, in TBST. Anti‐rabbit (Cell Signaling Technology, Danvers, MA, USA; cat. no. 7074) and anti‐mouse (Cell Signaling Technology, cat. no. 7076) secondary antibodies were used at a concentration of 1:10,000, in TBST. Antibodies were directed against: acetyl‐CoA carboxylase (ACC; Cell Signaling Technology, cat. no. 3662), p‐ACC^Ser79^ (Cell Signaling Technology, cat. no. 3661), DRP1 (Cell Signaling Technology, cat. no. 8570), p‐DRP1^Ser616^ (Cell Signaling Technology, cat. no. 3455), p‐DRP1^Ser637^ (Cell Signaling Technology, cat. no. 4867), MFF (Cell Signaling Technology, cat. no. 84580), p‐MFF^Ser146^ (Cell Signaling Technology, cat. no. 49281), and Parkin (Cell Signaling Technology, cat. no. 32833).

### 2.9 Statistical analysis

Sample size was determined from a power analysis, using a previously reported 30% increase in AMPK^Thr172^ phosphorylation immediately following exercise in the fasted state (Stocks et al., 2019). It was determined that eight participants in each condition were required to detect statistical significance (power = 80%, α = 0.05). Statistical analysis was performed using a two‐way repeated measures ANOVA to assess for effects of time, condition (Fed or Fasted) and time × condition interaction effects for all time‐course data. Where applicable, a Tukey *post hoc* test was used to assess for multiple comparisons. For Western blotting *n* = 8; except for Fed–Pre where due to insufficient sample volume, *n* = 7. As a result, a mixed‐effects analysis was applied to western blotting data, with Tukey *post hoc* testing still used to assess multiple comparisons. For data recorded only during the exercise period, Student's paired *t*‐test was used to compare for group differences (i.e., Fed vs. Fasted) for the mean values of gas exchange (${\dot{V}}_{O_{2}}$, ${\dot{V}}_{CO_{2}}$ and RER), RPE and HR. All statistical analysis was performed using GraphPad Prism version 10.0.0 for windows (GraphPad Software, Boston, MA, USA). Data are presented as means ± SD. Statistical significance was set as *P* ≤ 0.05.

## 3 RESULTS

### 3.1 The physiological response to exercise in the fed and fasted state

There was no significant difference reported in HR (*P* = 0.257) or RPE (*P* = 0.802) between fed or fasted groups during exercise (Tables 2 and 3, respectively). Similarly, there was no significant differences reported in mean values for ${\dot{V}}_{O_{2}}$ (L/min) (*P* = 0.520) or RER (${\dot{V}}_{CO_{2}}$/${\dot{V}}_{O_{2}}$) (*P* = 0.146) between fed and fasted groups during exercise (Table 4). The mean ${\dot{V}}_{CO_{2}}$ (L/min) during exercise in the fed group was significantly elevated (*P* = 0.037), when compared with that of the fasted group (Table 4). Elevated ${\dot{V}}_{CO_{2}}$ was not necessarily reflected in rates of substrate utilization (Table 5), where it was found that carbohydrate (*P* = 0.053) and fat oxidation (*P* = 0.126) did not significantly vary between fed and fasted groups during exercise.

**TABLE 2:** Heart rate (HR) measured at rest (0 min) and during exercise (15–60 min) under fed and fasted conditions.

		0 min	15 min	30 min	45 min	60 min	Mean	*P*
**HR (bpm)**	**Fed**	76 ± 9	165 ± 11	162 ± 15	164 ± 19	164 ± 18	164 ± 14	0.257
	**Fasted**	68 ± 12	161 ± 15	159 ± 18	157 ± 17	161 ± 17	160 ± 16	

*Note*: Data are presented as means ± SD (*n* = 8). Mean heart rate recorded during exercise (15–60 min) was compared between fed and fasted conditions using Student's paired *t*‐test.

**TABLE 3:** Rating of perceived exertion (RPE) measured at rest (0 min) and during exercise (15–60 min) under fed and fasted conditions.

		0 min	15 min	30 min	45 min	60 min	Mean	*P*
**RPE (6–20 Borg scale)**	**Fed**	—	13 ± 1	15 ± 2	16 ± 1	15 ± 1	15 ± 1	0.802
	**Fasted**	—	14 ± 2	15 ± 2	16 ± 2	15 ± 3	15 ± 12	

*Note*: Data are presented as means ± SD (*n* = 8). The mean RPE recorded during exercise (15–60 min) was compared between fed and fasted conditions using Student's paired *t*‐test.

**TABLE 4:** Gas Exchange values recorded at rest (0 min) and during exercise (15–60 min) under fed and fasted conditions.

		0 min	15 min	30 min	45 min	60 min	Mean	*P*
${\dot{V}}_{O_{2}}$ **(L/min)**	**Fed**	0.41 ± 0.05	2.28 ± 0.30	2.04 ± 0.44	1.98 ± 0.48	1.95 ± 0.42	2.06 ± 0.40	0.520
	**Fasted**	0.37 ± 0.1	2.10 ± 0.31	2.01 ± 0.43	1.93 ± 0.52	2.04 ± 0.51	2.02 ± 0.43	
${\dot{V}}_{CO_{2}}$ **(L/min)**	**Fed**	0.37 ± 0.07	2.25 ± 0.33	1.91 ± 0.42	1.81 ± 0.44	1.77 ± 0.40	1.94 ± 0.38	0.037
	**Fasted**	0.29 ± 0.05	2.00 ± 0.27	1.82 ± 0.32	1.65 ± 0.41	1.75 ± 0.43	1.80 ± 0.33	
**RER (** ${\dot{V}}_{CO_{2}}$ **/** ${\dot{V}}_{O_{2}}$)	**Fed**	0.92 ± 0.16	0.99 ± 0.05	0.94 ± 0.03	0.92 ± 0.03	0.91 ± 0.04	0.94 ± 0.03	0.146
	**Fasted**	0.85 ± 0.19	0.96 ± 0.04	0.92 ± 0.08	0.86 ± 0.05	0.87 ± 0.03	0.90 ± 0.04	

*Note*: Data are presented as mean ± SD (*n* = 8). Mean respiratory gas values recorded during exercise (15–60 min) were compared between fed and fasted conditions using Student's paired *t*‐test.

**TABLE 5:** Carbohydrate and fat oxidation rates at rest (0 min) and during exercise (15–60 min) under fed and fasted conditions.

		0 min	15 min	30 min	45 min	60 min	Mean	*P*
**Carbohydrate oxidation (g/min)**	**Fed**	0.40 ± 0.22	2.72 ± 0.60	2.01 ± 0.52	1.76 ± 0.50	1.67 ± 0.52	**2.04 ± 0.49**	0.053
	**Fasted**	0.16 ± 0.12	2.18 ± 0.36	1.73 ± 0.47	1.21 ± 0.39	1.36 ± 0.35	**1.62 ± 0.30**	
**Fat oxidation (g/min)**	**Fed**	0.08 ± 0.04	0.09 ± 0.14	0.20 ± 0.11	0.28 ± 0.12	0.30 ± 0.14	**0.22 ± 0.12**	0.126
	**Fasted**	0.13 ± 0.05	0.19 ± 0.13	0.33 ± 0.25	0.48 ± 0.25	0.47 ± 0.16	**0.36 ± 0.19**	

*Note*: Data are presented as mean ± SD (*n* = 8). Mean rates of substrate oxidation recorded during exercise (15–60 min) were compared between fed and fasted conditions using Student's paired *t*‐test.

A two‐way ANOVA identified a main effect of time (*P* < 0.0001) and a time × treatment interaction effect (*P* < 0.0001) on plasma glucose levels (Figure 1a). Tukey *post hoc* testing identified a significant increase (*P* = 0.0039) in plasma glucose in the fed group 20 min following consumption of the carbohydrate‐rich breakfast. Further, 15 min into exercise (135 min), plasma glucose concentrations in the fed group were significantly decreased (*P* = 0.0031) from baseline. Compared to the fasted group, the fed group displayed significantly greater and lower plasma glucose levels at 20 min (*P* = 0.0001) and 135 min (*P* < 0.0001), respectively. Plasma glucose levels were also significantly different between fed and fasted groups at 150 min (*P* = 0.0013), 200 min (*P* = 0.0186) and 360 min (*P* = 0.0293). A two‐way ANOVA showed a significant (*P* < 0.0001) effect of time on plasma lactate levels (Figure 1b). Plasma lactate levels were significantly increased (*P* < 0.0001) during the exercise period (135–180 min) and remained significantly elevated (*P* = 0.0062) 20 min into recovery (200 min), irrespective of group (i.e., Fed or Fasted).

**FIGURE 1:**
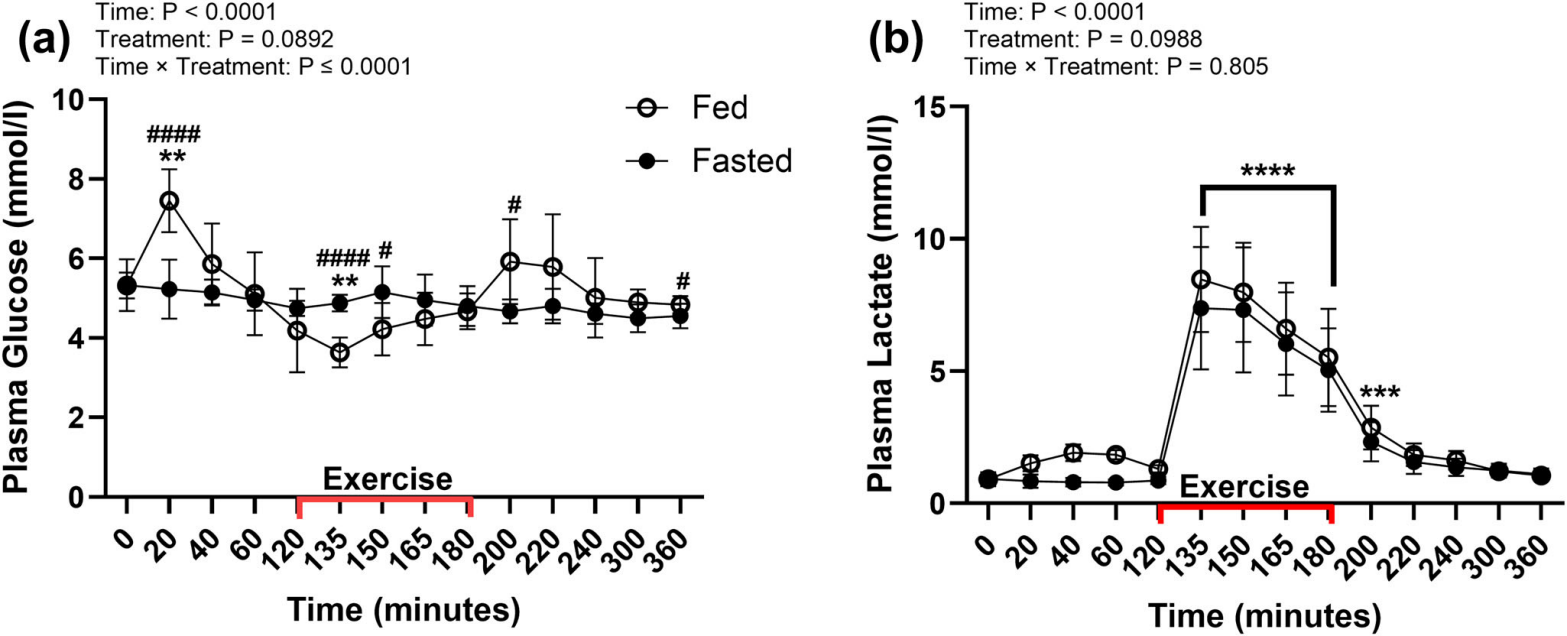
Time course data for the plasma concentrations of glucose (a) and lactate (b) with exercise performed between 120 and 180 min. Data are presented as means ± SD (*n* = 8). **P < 0.01, ***P ≤ 0.001, ****P ≤ 0.0001: significantly different from baseline sample (0 min, main effect of time). #*P* ≤ 0.05, ####*P* ≤ 0.0001: significantly different between fed and fasted conditions (interaction effect).

### 3.2 Confirming enrichment of mitochondrial and cytosolic cell fractions

Cytosolic and mitochondrial cell fractions were isolated from muscle biopsies taken from the vastus lateralis before (Pre), immediately after (Post), and 3 h following exercise. A high degree of enrichment was confirmed by the relative abundance of GAPDH and VDAC in the cytosolic and mitochondrial fractions, respectively, along with their very low concentration in the opposing fraction (Figure 2).

**FIGURE 2:**
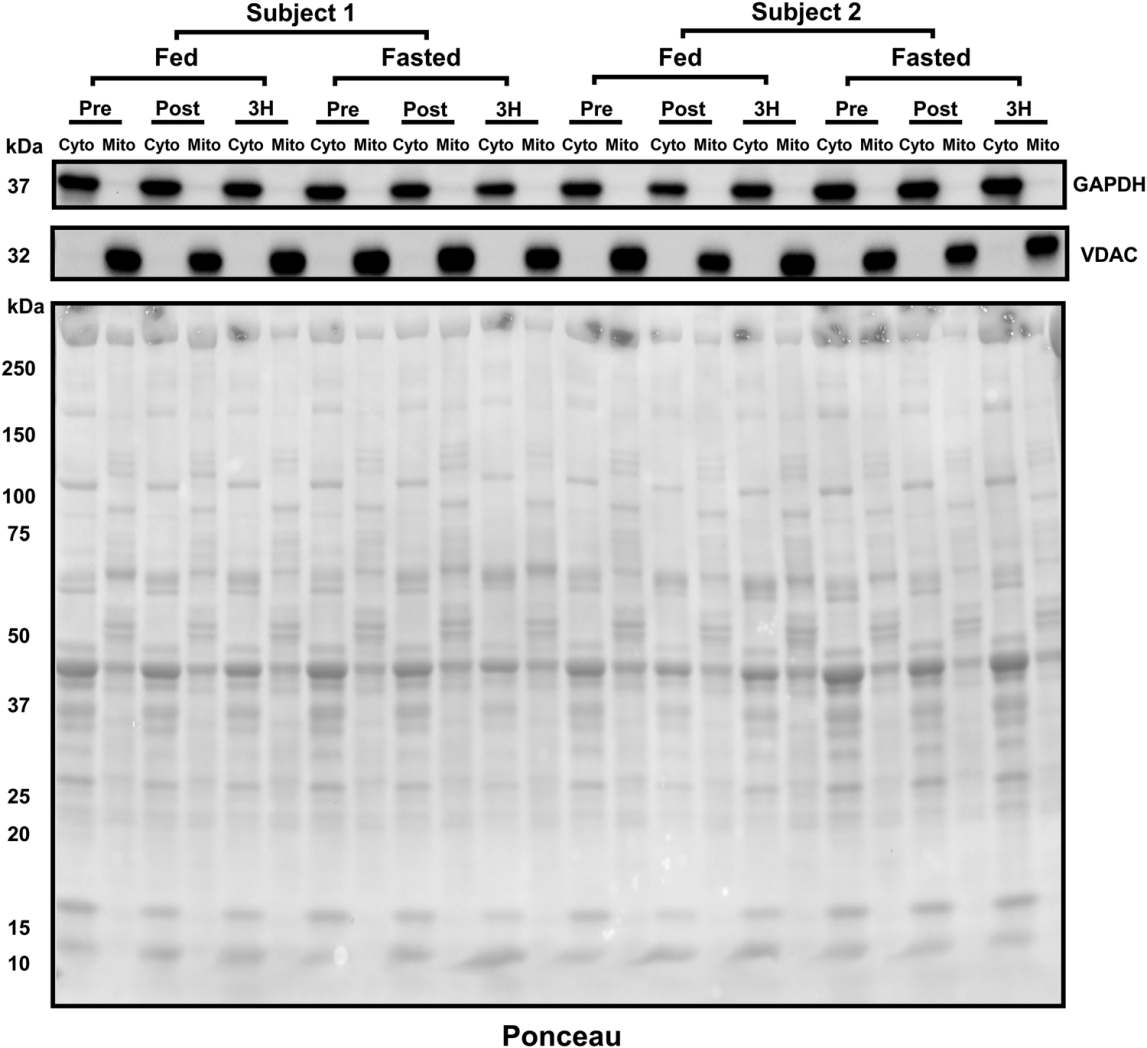
A western blot representative image displays the relative abundance of GAPDH and VDAC in the mitochondrial and cytosolic enriched cell fractions. Ponceau staining allowed for correction against total lane protein abundance.

### 3.3 Exercise in the fed and fasted state increased ACC^Ser79^ phosphorylation

Acetyl‐CoA carboxylase (ACC) subcellular localisation was unchanged with exercise (data not shown). Western blotting was used to probe for phosphorylated ACC ^Ser79^, a well‐established AMPK phosphorylation site (Galic et al., 2018). For cytosolic and mitochondrial ACC^Ser79^ phosphorylation (Figure 3a and b, respectively), a mixed‐effects analysis revealed a main effect of time (*P* = 0.0031 and *P* < 0.0001, respectively). *Post hoc* analysis revealed a significant 2‐fold increase (*P* = 0.0003) in cytosolic ACC^Ser79^ phosphorylation post‐exercise, in the fasted group. No significant differences were identified for cytosolic ACC^Ser79^ phosphorylation in the fed group immediately post or 3 h after exercise (*P* = 0.129 and *P* = 0.846, respectively). Mitochondrial ACC^Ser79^ phosphorylation was significantly increased 2.3‐ and 3‐fold post‐exercise in fed and fasted groups immediately after exercise (*P* = 0.0070 and *P* < 0.0001, respectively).

**FIGURE 3:**
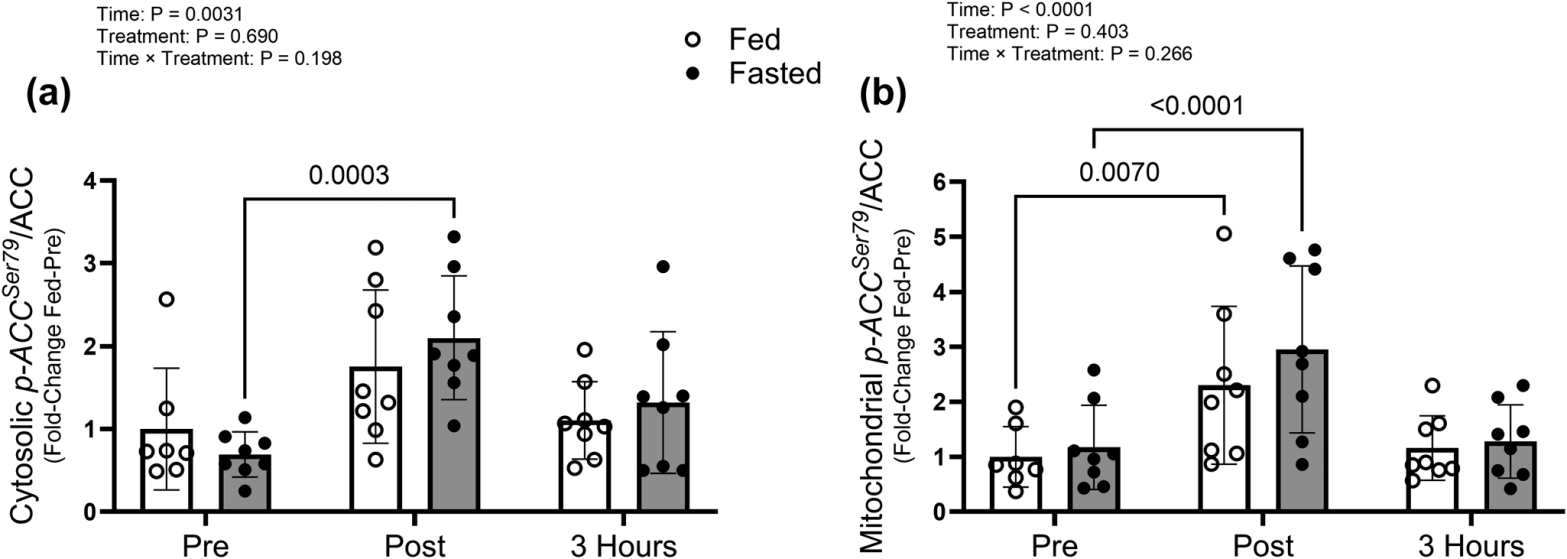
Cytosolic (a) and mitochondrial (b) phosphorylation of ACC^Ser79^ in cell fractions isolated from muscle biopsies taken from the vastus lateralis before (Pre), immediately after (Post) and 3 h following exercise. Data are presented as means ± SD with individual responses (*n* = 8; except Fed–Pre, *n* = 7).

### 3.4 Mitochondrial dynamics signalling following fed and fasted exercise

Cytosolic protein content of DRP1 (Figure 4a) was not affected by time (*P* = 0.214) following exercise in either the fed or the fasted group. A mixed‐effects analysis of mitochondrial DRP1 and the ratio of mitochondrial to cytosolic DRP1 (Figures 4b and c, respectively) identified a significant time × treatment effect (*P* = 0.0323 and *P* = 0.0309, respectively). Tukey's multiple comparison test did not reveal any significant differences (*P* > 0.05) between fed and fasted conditions in mitochondrial DRP1. When expressed as a ratio to cytosolic DRP1, mitochondrial DRP1 was significantly diminished to ∼0.4‐fold in the fed group 3 h post‐exercise (*P* = 0.0294) when compared to the fasted group.

**FIGURE 4:**
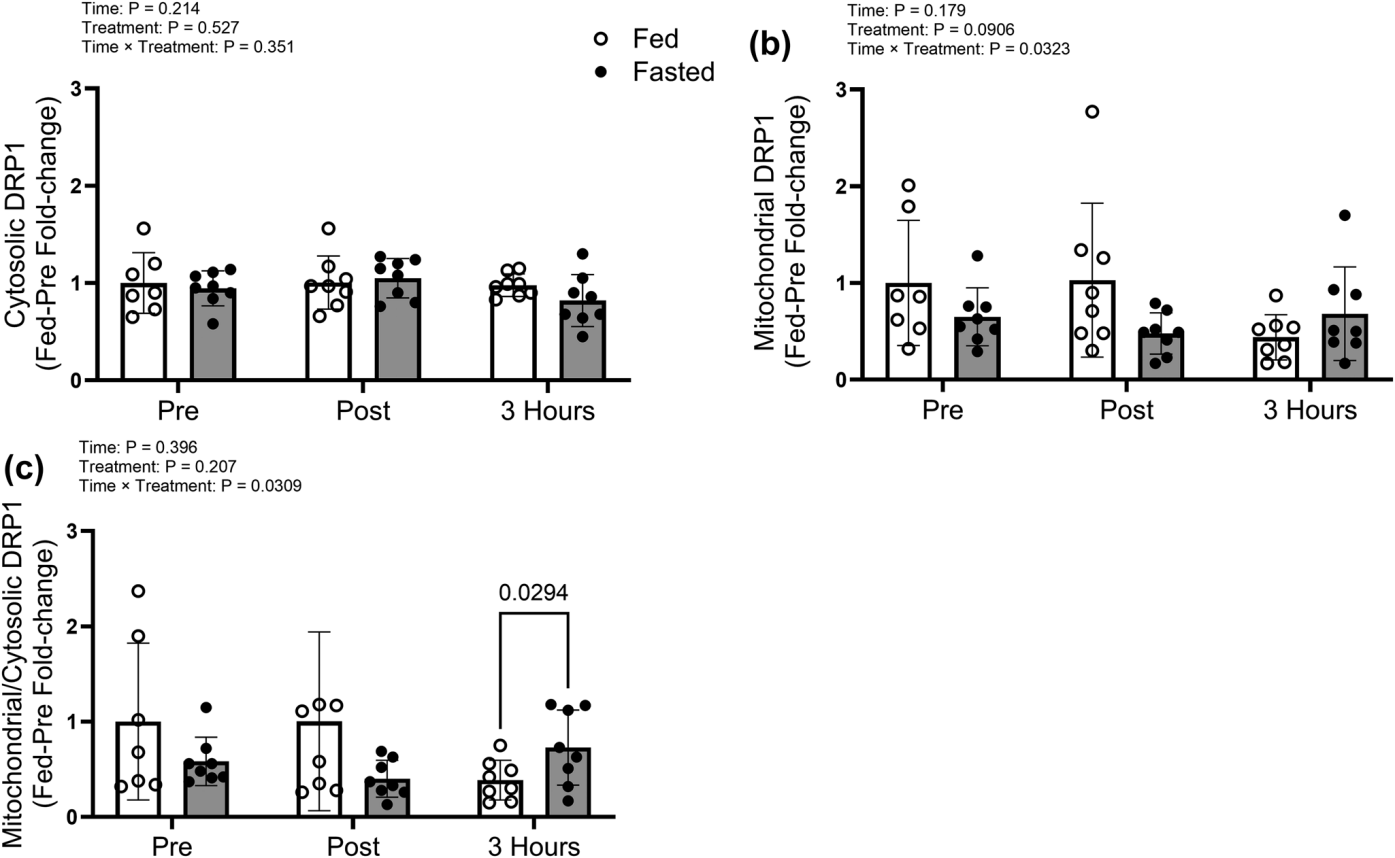
Cytosolic (a), mitochondrial (b), and the mitochondrial to cytosolic ratio (c) of DRP1 in cell fractions isolated from muscle biopsies taken from the vastus lateralis before (Pre), immediately after (Post) and 3 h following exercise. Data are presented as means ± SD with individual responses (*n* = 8; except Fed–Pre, *n* = 7).

The absolute cytosolic content of phosphorylated DRP1^Ser616^ (Figure 5a) was unchanged with time (*P* = 0.112) following exercise in either the fed or the fasted group. However, a mixed‐effects analysis of normalised cytosolic phosphorylated DRP1^Ser616^ (Figure 5b) identified a significant effect of time (*P* = 0.0480), whereby phosphorylated DRP1^Ser616^ was significantly increased ∼2‐fold (*P* = 0.0051), 3 h following exercise in the fasted group. In the mitochondrial fraction, the absolute and normalized content of phosphorylated DRP1^Ser616^ (Figure 5c and d, respectively) was unchanged with time (*P* = 0.782 and *P* = 0.286, respectively) following exercise in both the fed and the fasted group. A mixed‐effects analysis identified a main effect of time (*P* = 0.0322 and *P* = 0.0235, respectively) on the absolute and normalized cytosolic content of phosphorylated DRP1^Ser637^ (Figure 6a and 6b, respectively). However, Tukey's multiple comparison test revealed no significant (*P* > 0.05) differences in cytosolic DRP1^Ser637^ immediately following or 3 h post‐exercise in either the fed or the fasted group. A significant time × treatment interaction effect (*P* = 0.0061) was observed on normalized mitochondrial content of phosphorylated DRP1^Ser637^ (Figure 6d). *Post hoc* testing showed a significant (*P* = 0.0064) 2‐fold increase in mitochondrial phosphorylated DRP1^Ser637^ 3 h following exercise in the fed group. Increased mitochondrial content of phosphorylated DRP1^Ser637^ in the fed group was significantly greater (*P* = 0.0389) than in the fasted group at 3 h post‐exercise, also (Figure 6d).

**FIGURE 5:**
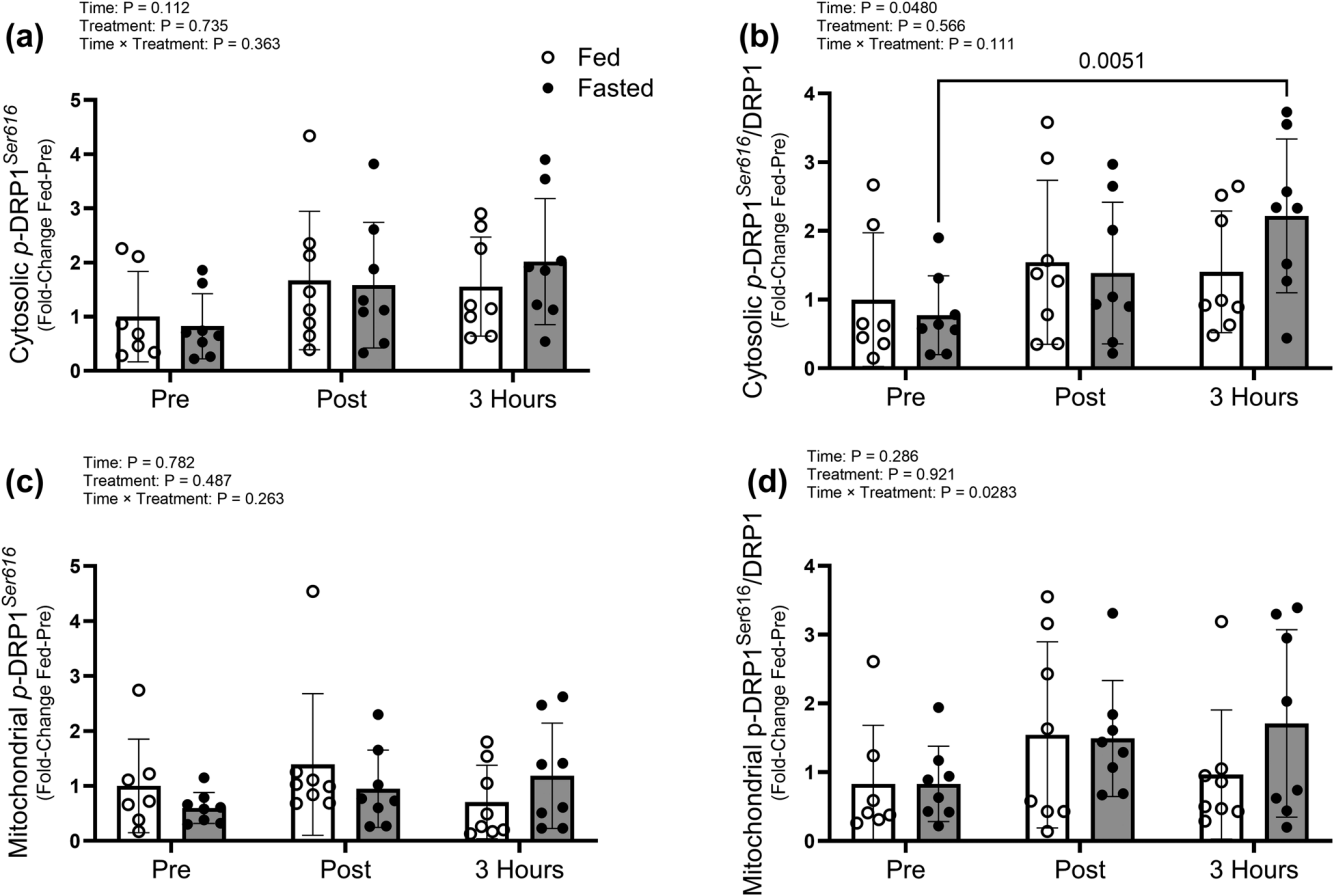
Absolute (a) and normalized (b) cytosolic content of phosphorylated DRP1^Ser616^ and absolute (c) and normalized (d) mitochondrial content of phosphorylated DRP1^Ser616^. Cell fractions were isolated from muscle biopsies taken from the vastus lateralis before (Pre), immediately after (Post) and 3 h following exercise. Data are presented as means ± SD with individual responses (*n* = 8; except Fed–Pre, *n* = 7).

**FIGURE 6:**
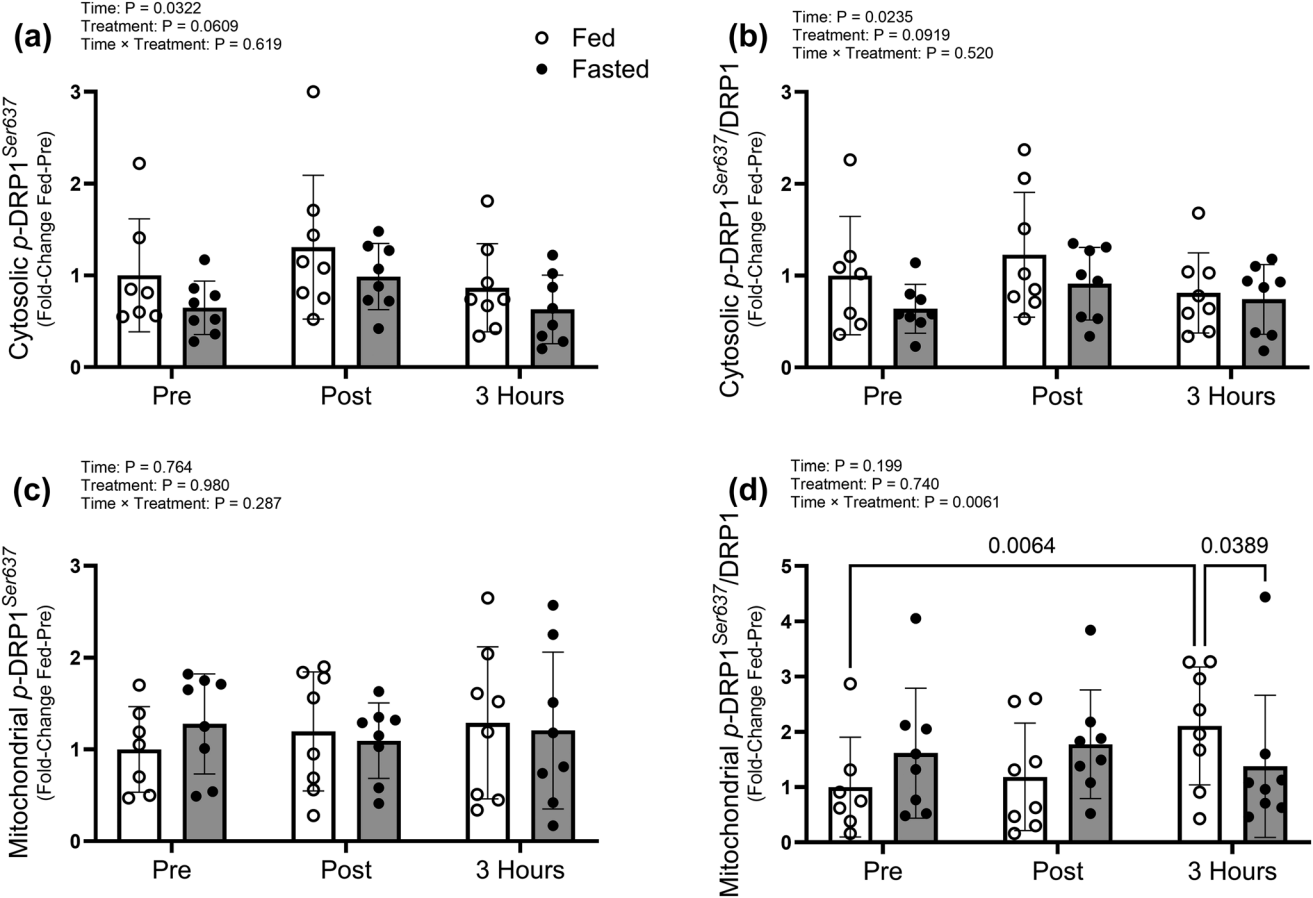
Absolute (a) and normalized (b) cytosolic content of phosphorylated DRP1^Ser637^ and absolute (c) and normalized (d) mitochondrial content of phosphorylated DRP1^Ser637^. Cell fractions were isolated from muscle biopsies taken from the vastus lateralis before (Pre), immediately after (Post) and 3 h following exercise. Data are presented as means ± SD with individual responses (*n* = 8; except Fed–Pre, *n* = 7).

A mixed‐effects analysis revealed no significant effect of time on the cytosolic (*P* = 0.119), mitochondrial (*P* = 0.0616), and ratio of mitochondrial to cytosolic (*P* = 0.227) MFF protein content (Figure 7a–c) with exercise irrespective of group (i.e., Fed or Fasted). Additionally, the absolute and normalized content of phosphorylated MFF^Ser146^ was unchanged with time (*P* > 0.05) in the cytosol and mitochondria (Figure 8a–d) following exercise in both the fed and the fasted group.

**FIGURE 7:**
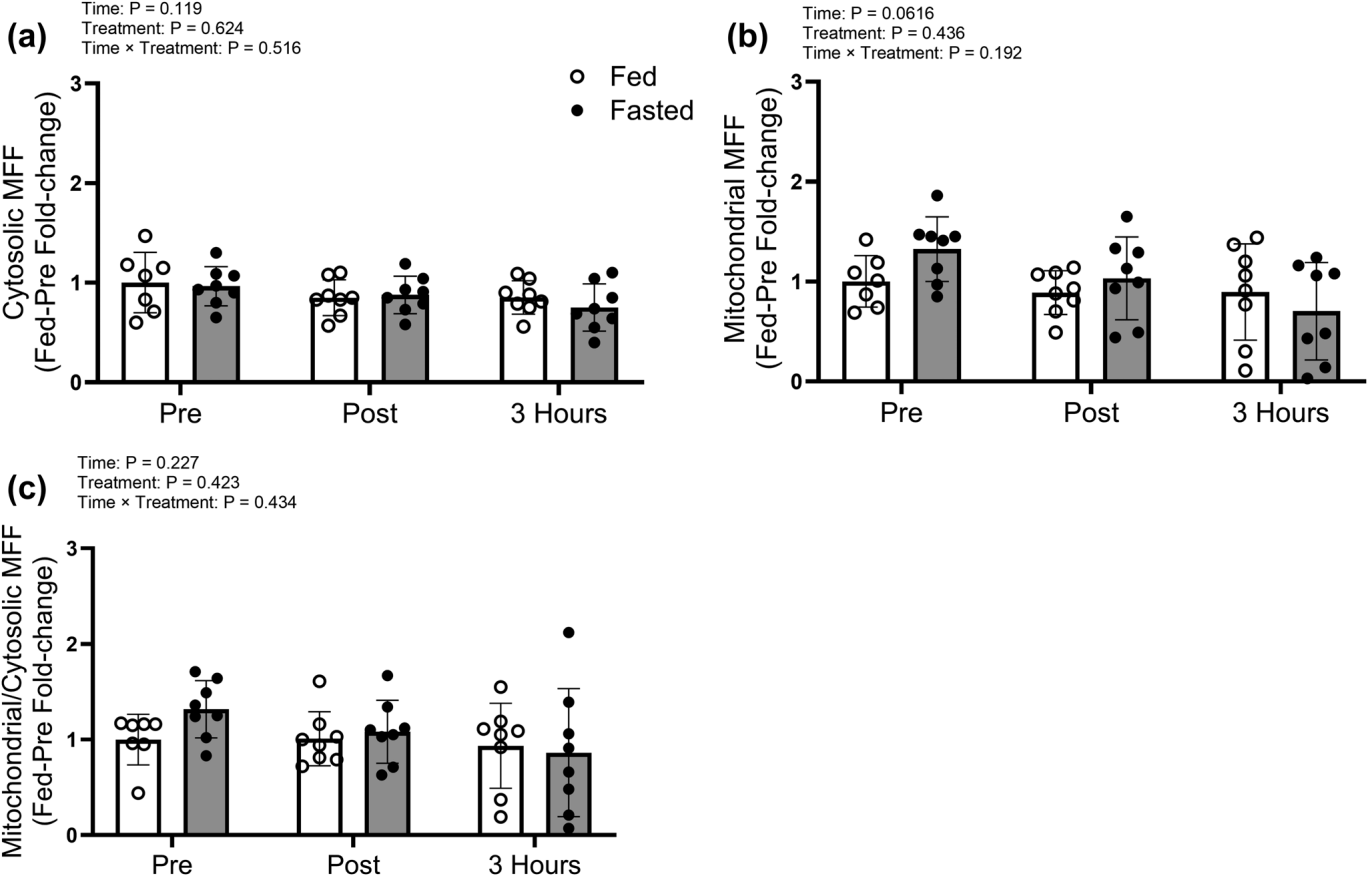
Cytosolic (a), mitochondrial (b), and the mitochondrial to cytosolic ratio (c) of MFF in cell fractions isolated from muscle biopsies taken from the vastus lateralis before (Pre), immediately after (Post), and 3 h following exercise. Data are presented as means ± SD with individual responses (*n* = 8; except Fed–Pre, *n* = 7).

**FIGURE 8:**
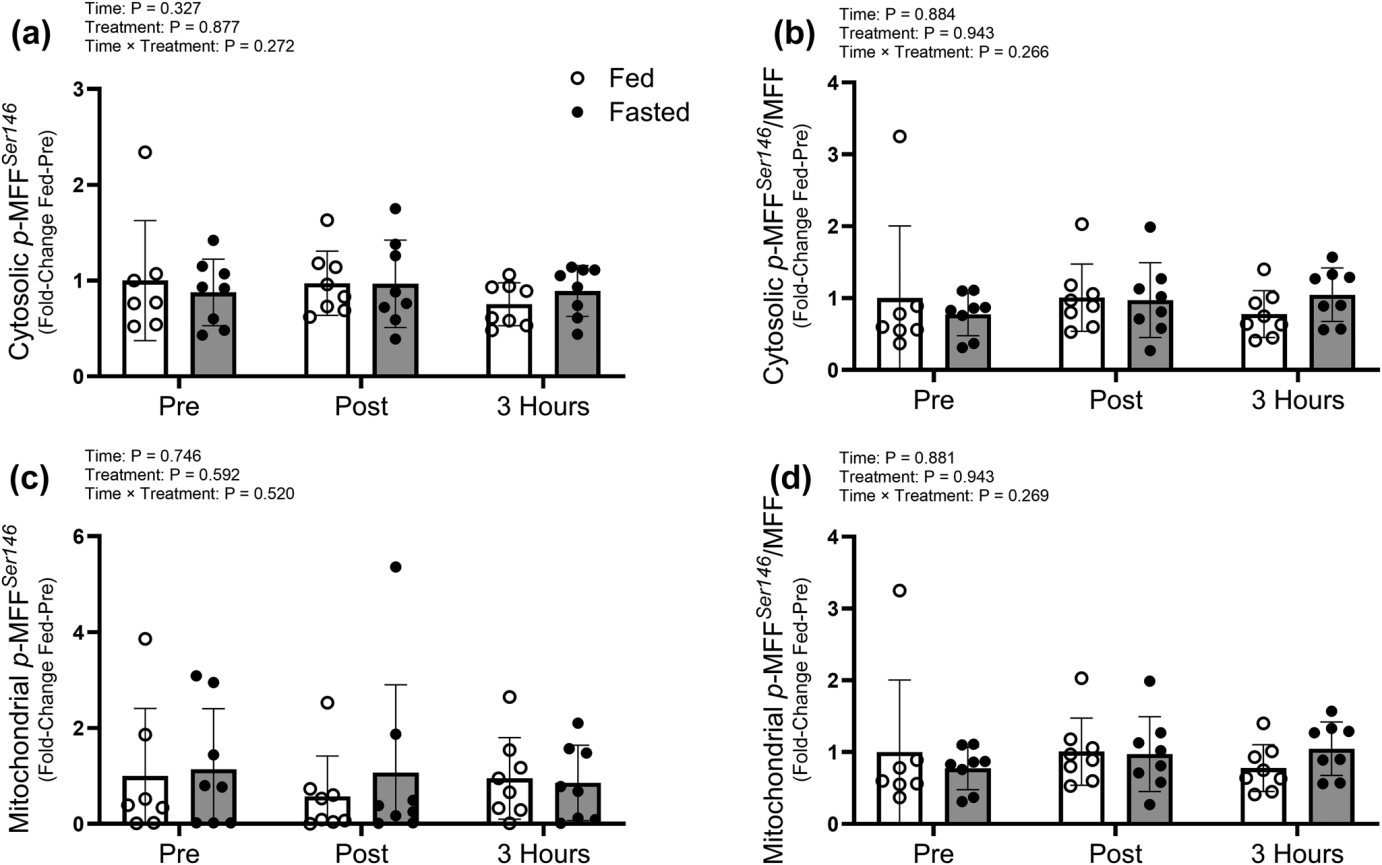
Absolute (a) and normalized (b) cytosolic content of phosphorylated MFF^Ser146^ and absolute (c) and normalized (d) mitochondrial content of phosphorylated MFF^Ser146^. Cell fractions were isolated from muscle biopsies taken from the vastus lateralis before (Pre), immediately after (Post) and 3 h following exercise. Data are presented as means ± SD with individual responses (*n* = 8; except Fed–Pre, *n* = 7).

Western blotting was used to probe for the protein content of Parkin in cytosolic and mitochondrial fractions. No change with time (*P* = 0.760) in cytosolic Parkin content (Figure 9) was observed following exercise in both the fed and fasted groups. Further, an absence of Parkin in mitochondrial fractions was observed pre‐ and post‐exercise (Figure 10).

**FIGURE 9:**
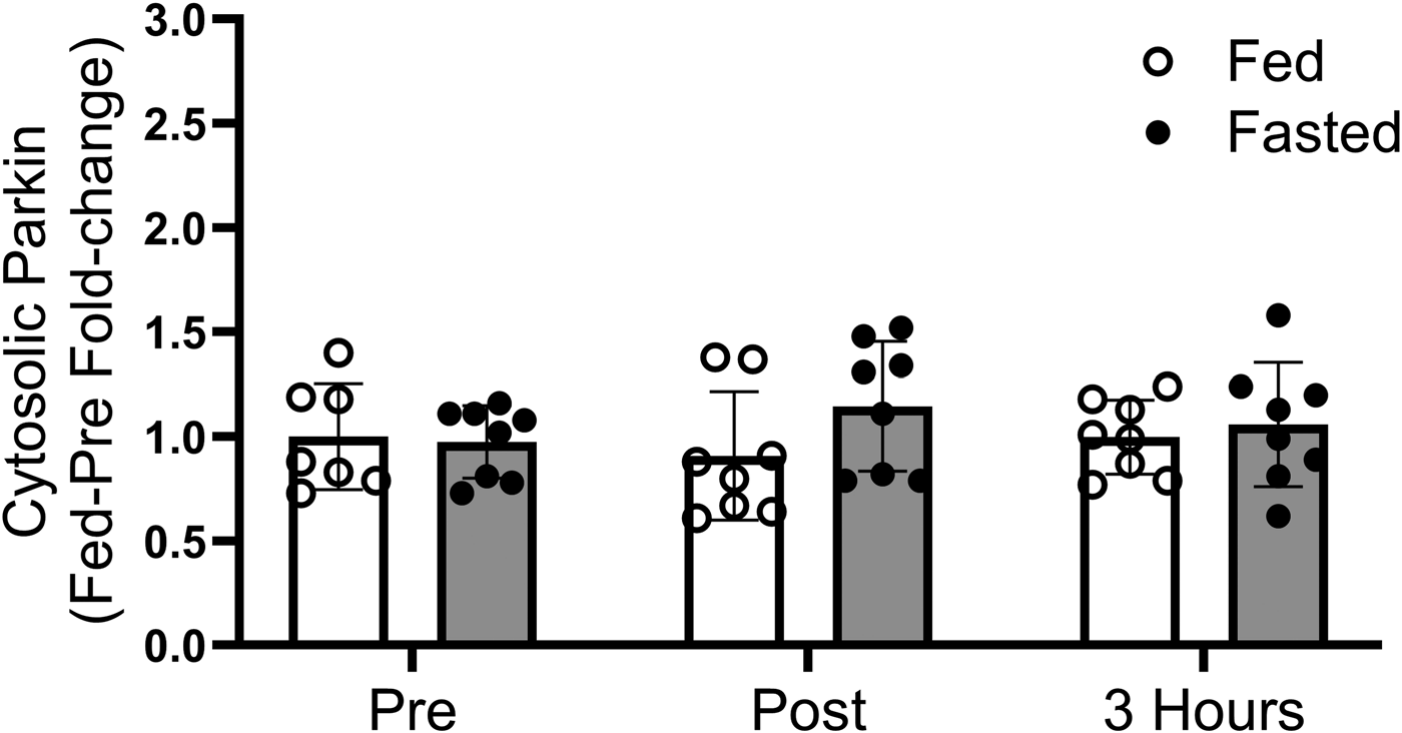
Cytosolic Parkin in cell fractions isolated from muscle biopsies taken from the vastus lateralis before (Pre), immediately after (Post) and 3 h following exercise. Data are presented as means ± SD with individual responses (*n* = 8; except Fed–Pre, *n* = 7).

**FIGURE 10:**
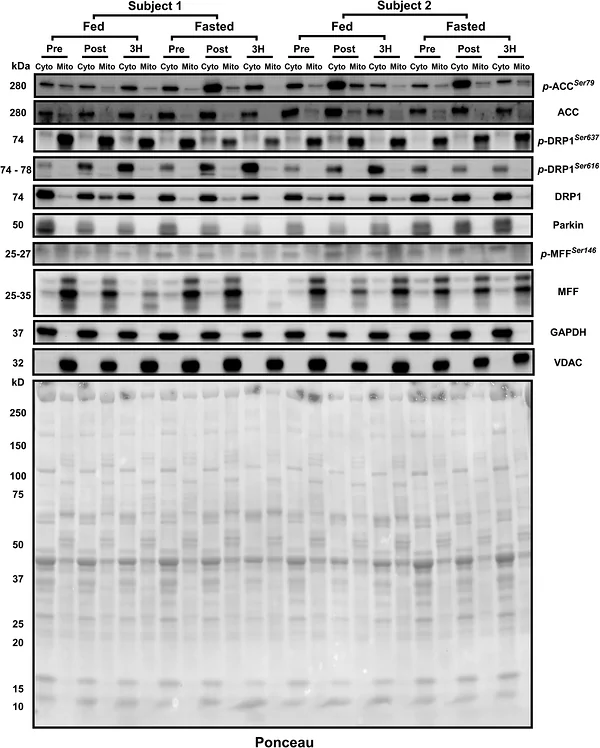
Representative images of the western blot data presented in Figures 4, 5, 6, 7, 8, 9, 10. Western blotting was carried out in cell fractions isolated from muscle biopsies taken from the vastus lateralis.

## 4 DISCUSSION

The overall aim of this study was to investigate the mitochondrial dynamics signalling response to a bout of acute aerobic exercise in the fed or fasted state. Among the major results, an increase in mitochondrial ACC^Ser79^ phosphorylation was observed, indicating that the stimulus of exercise undertaken in both the fed and fasted conditions induced metabolic perturbations. Furthermore, there was an increase in cytosolic ACC^Ser79^ phosphorylation post‐exercise in the fasted group, indicating a potential augmentation of these exercise‐induced perturbations. Discrepancies in the mitochondrial abundance of DRP1 between fed and fasted groups post‐exercise were also found, suggesting a possible substrate‐sensitive modulation in the mitochondrial dynamics signalling response to exercise. Collectively, these data provide an insight into how altered substrate availability influences the mitochondrial dynamics signalling response with exercise and highlight how altered stress signalling may underpin this response.

### 4.1 Physiological responses to exercise in the fed and fasted state

Physiological measurements during exercise such as HR and RPE did not differ between fed and fasted groups. Similarly, there was no difference between fed and fasted groups for ${\dot{V}}_{O_{2}}$ or RER. However, it was observed that ${\dot{V}}_{CO_{2}}$ was significantly increased during exercise in the fed group when compared with that of the fasted group. Although stoichiometry of glucose oxidation shows a greater proportionate production of CO_2_ relative to the oxidation of palmitate (Jeukendrup & Wallis, 2005), this did not manifest as group differences in rates of substrate utilization. No significant differences between fed and fasted groups were observed when deriving rates of carbohydrate or fat oxidation based on ${\dot{V}}_{CO_{2}}$ and ${\dot{V}}_{O_{2}}$. Plasma glucose was significantly diminished 15 min (135 min) into exercise in the fed condition. In this regard, exercise has been shown to increase glucose uptake in an insulin‐independent manner through increasing translocation of GLUT4 receptors to the sarcolemma (Goodyear & Kahn, 1998). The reduction in plasma glucose at the onset of exercise in the fed group could therefore result from a synergistic combination of insulin‐dependent and insulin‐independent glucose uptake. However, as the present work did not measure plasma insulin directly, only a limited interpretation can be made about the contribution of insulin‐dependent and insulin‐independent glucose uptake mechanisms. Additionally, increased glucose utilisation was not suggested by group differences in plasma lactate measurements, which were found to significantly increase in both fed and fasted groups during exercise.

### 4.2 Exercise in the fed and fasted state increased ACC^Ser79^ phosphorylation

ACC^Ser79^ is a direct substrate of AMPK which has been shown to be phosphorylated in response to exercise, promoting a shift towards increased fatty acid oxidation (Chen et al., 2000). Mitochondrial ACC^Ser79^ phosphorylation significantly increased ∼2‐fold post‐exercise in both fed and fasted groups, indicating that the bout of acute exercise induced metabolic perturbations. Interestingly, cytosolic ACC^Ser79^ phosphorylation was only elevated in the fasted group post‐exercise. AMPK has been shown to directly phosphorylate ACC^Ser79^ in response to fasting, thereby inhibiting fatty acid synthesis and promoting fatty acid oxidation (Galic et al., 2018). Previously reported increases in AMPK^Thr172^ phosphorylation with fasted exercise (compared to the same bout of exercise in the fed state) (Stocks et al., 2019) could potentially form the mechanistic basis for the observed increases in cytosolic ACC^Ser79^ phosphorylation in the fasted group. Nutrient deprivation has also been shown to increase formation of the AMPK activator AMP (de Lange et al., 2006). Additionally, AMPK has a regulatory glycogen binding domain with higher glycogen branch points allosterically inhibiting AMPK activation (McBride et al., 2009). In this regard, ACC^ser79^ phosphorylation reflects a combination of allosteric mechanisms and changes in phosphorylation which alter AMPK activity (Rothschild et al., 2022). Collectively, this highlights the potential for performing a given bout of moderate intensity aerobic exercise in the fasted state for the stress‐dependent enhancement of the signalling response in the cytosol. A previous meta‐analysis found that fasted exercise augmented rates of fat metabolism and associated molecular pathways (Aird et al., 2018). In the present study, increased cytosolic ACC^Ser79^ phosphorylation with fasted exercise was not accompanied by augmented rates of fat oxidation. Previous work of Chen and colleagues has convincingly highlighted the potential for there to be an uncoupling between ACC^Ser221^ phosphorylation and rates of substrate oxidation during a graded progressive‐intensity exercise protocol (Chen et al., 2003). Consistent with the work of McConell suggesting that ACC phosphorylation and rates of fat oxidation may be uncoupled during exercise (McConell, 2020), the present study demonstrates this may be particularly relevant when considering compartment‐specific cytosolic ACC^Ser79^ phosphorylation.

### 4.3 The effect of fed and fasted exercise on mitochondrial dynamics signalling

Previous research has highlighted the potential for acute exercise to induce pro‐fission signalling in skeletal muscle. Specifically, work in murine reporter models has highlighted that elevated DRP1^Ser616^ phosphorylation following exercise coincides with the appearance of discrete punctuate mitolysosomes, suggesting that mitophagy was facilitated through increased mitochondrial fission (Laker et al., 2017). Previous studies in human skeletal muscle have also reported that bouts of submaximal acute aerobic exercise elicit increases in DRP1^Ser616^ phosphorylation (Kruse et al., 2016; Schwalm et al., 2017), a modification thought to increase mitochondrial fission. In the present study, mitochondrial, cytosolic and the ratio of mitochondrial to cytosolic DRP1 protein content was unchanged with exercise in both fed and fasted conditions. Collectively, these observations likely indicate a lack of a mitochondrial fission signalling response following a bout of submaximal aerobic exercise in recreationally active men. Previous in vitro and murine models reporting increased DRP1 translocation upon mitochondrial stress have used supraphysiological stimuli such as pharmacological agents and models of exhaustive exercise, respectively (Hu & Qi, 2020; Vainshtein et al., 2015). In this regard, it is possible that a submaximal steady‐state bout of aerobic exercise at 50–70% *W*_max_ was insufficient to elicit the same response. Previous human studies reporting increased DRP1^Ser616^ phosphorylation did also not examine mitochondrial DRP1 translocation directly (Kruse et al., 2016; Schwalm et al., 2017), making it difficult to corroborate that increased fission signalling led to meaningful changes in DRP1 localisation following aerobic exercise. While the absence of changes in subcellular DRP1 observed here suggests a lack of a mitochondrial fission response with exercise, it is important to consider that subcellular protein localisation is only a surrogate measure of DRP1 activity. Therefore, direct measurements of GTPase activity (Ingerman & Nunnari, 2005) would be required to definitively rule out a lack of a mitochondrial fission response.

Interestingly, however, a divergence in DRP1 phosphorylation between fed and fasted groups emerged post‐exercise and potentially coincided with group differences in DRP1 localisation. Specifically, 3 h post‐exercise, there was a significant ∼2.2‐fold increase in cytosolic DRP1^Ser616^ phosphorylation in the fasted group only. As mentioned, DRP1^Ser616^ phosphorylation is considered pro‐fission and is associated with translocation of DRP1 to the mitochondria (Hu et al., 2017). Accordingly, DRP1^Ser616^ phosphorylation (or lack thereof) may account for the significant ∼0.6‐fold decrease in the ratio of mitochondrial to cytosolic DRP1 protein content that was observed 3 h post‐exercise in the fed group. This highlights the potential for substrate availability to influence the mitochondrial dynamics signalling response, particularly when considering that increased DRP1^Ser616^ phosphorylation following fasted exercise coincided with greater stress signalling in the same subcellular compartment (i.e., increased cytosolic ACC^Ser79^ phosphorylation). While previous human studies utilised aerobic exercise protocols of greater duration and intensity (i.e., 120 mins at 70% of ${\dot{V}}_{O_{2}\max}$) (Schwalm et al., 2017) or recruited participants with a greater average BMI and age (age: 54.7 ± 2.3 years; BMI: 29.0 ± 0.9 kg/m^2^) (Kruse et al., 2016), thereby limiting direct comparisons with this study. It should be noted that Schwalm and colleagues, using a similar model of fed–fasted exercise reported a significant increase cytosolic DRP1^Ser616^ phosphorylation 1 h following fasted exercise, whereas no increase was seen following fed exercise (Schwalm et al., 2017). Phosphorylation of DRP1 at Ser637 has been previously shown to be associated with repression of mitochondrial translocation of DRP1 (Cereghetti et al., 2008; Zhang et al., 2016). In this work we show the presence of DRP1^Ser637^ in mitochondrial fractions and that mitochondrial DRP1^Ser637^ phosphorylation was significantly increased ∼2‐fold 3 h post‐exercise in the fed group only. This is in support of previous in vitro work by Yu and colleagues, who demonstrated that phosphorylated DRP1^Ser637^ still retains the capacity to transition between the cytosol and mitochondria, with phosphorylation at this residue thought to influence the association of DRP1 with adaptors, such as MFF (Yu et al., 2019). Differences between fed and fasted groups in the post‐exercise mitochondrial dynamics signalling response did not implicate the DRP1 adaptor MFF, as no changes were observed in subcellular localisation or Ser146 phosphorylation.

### 4.4 Parkin was not observed in mitochondrial fractions following exercise

Previous studies have reported that Parkin protein content is increased in skeletal muscle following chronic exercise (Arribat et al., 2019; Balan et al., 2019; Tarpey et al., 2017). However, less is known about the acute molecular response and processes of Parkin activation. Following continuous moderate intensity aerobic exercise in trained athletes, the protein content of Parkin in mitochondrial fractions has been reported to be unchanged (Schwalm et al., 2017). Conversely, it has been reported in whole cell lysates that acute resistance exercise induces a decrease in total Parkin expression (Díaz‐Castro et al., 2024). Here we report that cytosolic Parkin protein content was unchanged with both fed and fasted exercise. Parkin was not observed in mitochondrial fractions before or following exercise, irrespective of fed or fasted condition. These data collectively indicate that the subcellular localisation of Parkin is unchanged with moderate intensity fed and fasted aerobic exercise. The previously reported presence of Parkin in mitochondrial fractions isolated from human skeletal muscle (Schwalm et al., 2017) may owe to methodological discrepancies. Despite confirmation of cell fraction purity in that study (Schwalm et al., 2017), the high cytosolic abundance of Parkin means that even minimal contamination could give the presence of a false enrichment. Alternatively, the amount of mitochondrial protein loaded (10 µg) may not be sufficient to immunoblot for Parkin, when considering the low abundance and potential for protein loss during the fractionation process (Baghirova et al., 2015).

### 4.5 Conclusion

Differences in the localisation of total DRP1, DRP1^Ser616^ and DRP1^Ser637^ phosphorylation between fed and fasted conditions highlight the potential for acute alterations in substrate availability to potentially influence the mitochondrial dynamics signalling response with exercise. Based on post‐exercise changes in ACC phosphorylation, findings from this study indicate that the cytosolic stress signalling response is potentially enhanced with fasted exercise, in accordance with previously reported studies (Aird et al., 2018; Stocks et al., 2019). These results further elucidate the influence of exercise on mitochondrial dynamics and contribute to an evolving perspective on how exercise derives benefits for skeletal muscle health through eliciting mitochondrial adaptations. Surprisingly, in contrast to our hypothesis, a lack of a mitochondrial fission signalling response was observed post‐exercise. It is well‐documented that there are sex differences in the physiological and molecular response to both acute and chronic exercise (Landen et al., 2023). The findings presented here may therefore diverge from the response that would be observed in females. Future studies should therefore utilise female participants to determine the bearing that biological sex has on the mitochondrial dynamics signalling response with exercise. Future studies should also consider examining DRP1 signalling through markers such as the formation of tetramer species (Hu & Qi, 2020), alongside the subcellular localisation presented here. Studies which take these methodological refinements and the influence of variables such as biological sex into consideration will be well positioned to further investigate the potential for acute alterations in substrate availability to influence the mitochondrial dynamics signalling response with exercise.

## AUTHOR CONTRIBUTIONS

Elya J. Ritenis, Andrew Philp and Donny M. Camera contributed to conception or design of the work; Elya J. Ritenis, Christos G. Stathis, Matthew B. Cooke, Stephen Keenan, Andrew Philp and Donny M. Camera performed acquisition, analysis, or interpretation of data for the work; and drafted or revised the work for critically important intellectual content. All authors have read and approved the final version of this manuscript and agree to be accountable for all aspects of the work in ensuring that questions related to the accuracy or integrity of any part of the work are appropriately investigated and resolved. All persons designated as authors qualify for authorship, and all those who qualify for authorship are listed.

## CONFLICT OF INTEREST

None declared.

## FUNDING INFORMATION

E.J.R. is supported by an Australian Government Research Training Program (RTP) Scholarship. The authors have no other funding sources to declare.

## GENERATIVE AI STATEMENT

No generative AI tools were used in the preparation of this manuscript.

## Data Availability

Source data for this study are not publicly available due to privacy and ethical restrictions, as collected data is private in nature. The source data are available to verified researchers upon request by contacting the corresponding author.
